# Mindfulness‐Based Eating Solution (MBES) for Body Dissatisfaction and Disordered Eating Attitudes in Nutrition Students During the COVID‐19 Pandemic: A Randomised Clinical Trial

**DOI:** 10.1111/jhn.70040

**Published:** 2025-03-17

**Authors:** Taísa Alves Silva, Amanda Thaís Flôres, Thamara Smaniotto Buttros, João Henrique Fabiano Motarelli, Geórgia das Graças Pena, Fernanda Rodrigues de Oliveira Penaforte, Camila Cremonezi Japur

**Affiliations:** ^1^ Programa de Pós‐Graduação em Nutrição e Metabolismo. Departamento de Ciências da Saúde Faculdade de Medicina de Ribeirão Preto, Universidade de São Paulo Ribeirão Preto São Paulo Brazil; ^2^ Núcleo de Estudos, Pesquisa e Extensão em Obesidade e Comportamento Alimentar, Faculdade de Medicina de Ribeirão Preto, Universidade de São Paulo (NEPOCA‐FMRP‐USP) Ribeirão Preto São Paulo Brazil; ^3^ Scuola di Agraria e Medicina Veterinaria Università degli Studi di Padova Padova Italy; ^4^ Nutricionista Clínica Ribeirão Preto São Paulo Brazil; ^5^ Instituto Consciência Alimentar São Paulo São Paulo Brazil; ^6^ Universidade Federal de Uberlândia Uberlândia Minas Gerais Brazil; ^7^ Programa de Pós‐Graduação em Psicologia / Departamento de Nutrição Universidade Federal do Triângulo Mineiro Uberaba Minas Gerais Brazil

**Keywords:** body dissatisfaction, COVID‐19, eating behaviour, mindful eating, mindfulness

## Abstract

**Background:**

Nutrition students are at greater risk of developing disordered eating attitudes, especially during the COVID‐19 pandemic. Mindfulness‐based interventions (MBIs) have proven to be beneficial in improving mental health outcomes and are also applied to issues related to food through mindful eating. The study aimed to evaluate the effect of a MBI on levels of body dissatisfaction and disordered eating attitudes among nutrition students during the COVID‐19 pandemic.

**Methods:**

This randomised clinical trial performed the ‘Mindfulness‐Based Eating Solution’ (MBES) intervention in nutrition students. Seventy‐eight adult women were randomised into the intervention group (*n* = 38) and control group (*n* = 40). The intervention group received eleven weekly sessions of MBES and two follow‐up sessions. Body dissatisfaction and appreciation, disordered eating attitudes, intuitive eating and mindfulness were assessed four times (at baseline, post‐intervention, 1‐month follow‐up and 3‐month follow‐up).

**Results:**

The intervention group showed decreased levels of body dissatisfaction (from 86.60 ± 6.13 to 64.13 ± 2.82) and disordered eating attitudes (from 1.46 ± 0.08 to 1.15 ± 0.02), and higher levels of reliance on hunger and satiety cues and mindfulness after the MBES intervention. Body dissatisfaction and levels of reliance on hunger and satiety cues were maintained at follow‐up, whereas the disordered eating attitudes and levels of mindfulness increased in the first and third months, respectively. No significant changes were found in the outcomes evaluated in the control group.

**Conclusions:**

The results suggest the positive effects of a MBI on the nutrition students’ body image perceptions and eating attitudes. Further research is needed to investigate such benefits and understand the related mechanisms in other populations.

## Summary

- Body dissatisfaction and disordered eating attitudes decreased after MBES intervention.
- Mindfulness and reliance on hunger and satiety cues levels increased after MBES intervention.
- Body dissatisfaction and reliance on hunger and satiety cues levels remained stable in the follow‐up period.
- Disordered eating attitudes and mindfulness increased in the follow‐up period.
- Body appreciation did not change after the intervention and during the follow‐up period.

## Graphical abstract

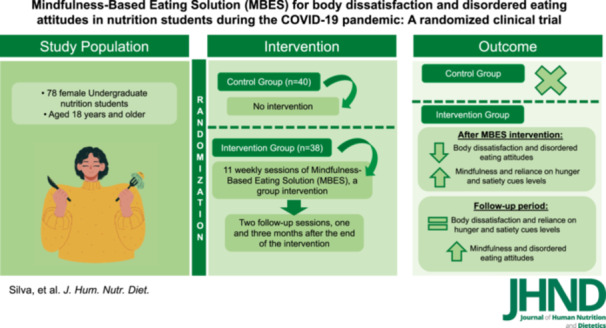

## Introduction

Lockdowns and isolation measures implemented during the COVID‐19 pandemic [1] increased exposure to social media, the idealisation of thinness and physical inactivity [2, 3]. These factors contributed to issues related to body weight and the development of disordered eating attitudes, particularly among high‐risk groups, such as young women [4].

Nutrition students, a predominantly female population of college students, experience significant social pressure to fit the thin body ideal and serve as role models for healthy eating [5]. However, it remains unclear whether the prevalence of body dissatisfaction and disordered eating among nutrition students differs from that observed in students from other fields, as some studies report higher rates [6, 7], whereas others indicate similar rates [8], or from that observed in the general population [9].

Therefore, interventions aimed at managing dysfunctional behaviours related to body image and eating are particularly important in this population, especially during the pandemic. Due to social distancing during the COVID‐19 pandemic, online interventions were favoured over face‐to‐face interventions, with online health interventions being used as a resource, such as mindfulness‐based interventions (‍MBIs). Mindfulness is characterised by the ability to be attentive to experiences, thoughts and emotions in the present moment, in a nonjudgemental manner [10]. Its cultivation may be related to the processes of self‐regulation, which integrates the control of attention and emotional regulation, stimulating behaviour changes [11].

Evidence suggests that MBIs can be beneficial in improving mental health outcomes, leading to reductions in anxiety, stress and depression in both face‐to‐face [12, 13] and online [14] interventions. Although mindfulness is not focused on helping people achieve their ideal body, research suggests that mindfulness levels are inversely related to body dissatisfaction [13, 15]. Mindfulness enhances present‐moment awareness, reducing body comparison and checking, thereby increasing body satisfaction and encouraging body acceptance [13]. Additionally, it heightens awareness of bodily sensations, emotions and thoughts, aiding in the identification of maladaptive patterns that contribute to body dissatisfaction [15, 16].

In addition, MBIs have been applied to issues related to food, through mindful eating—the ability to be aware of the physical and emotional sensations aroused during the act of eating or in a food‐related context, without judgement or criticism [17]. Currently, some protocols help train mindful eating skills, such as Mindfulness‐Based Eating Awareness Training (MB‐EAT) and the Mindfulness‐Based Eating Solution (MBES).

MB‐EAT promotes appetite regulation through awareness of internal body states versus external triggers to eat and self‐regulation theory that acknowledges the complex interplay of physiological and psychological processes [18]. MB‐EAT programme has shown effects in reducing frequency and severity of binge eating episodes in women with overweight and obesity, and binge eating disorder [19, 20], positive results for symptoms of depression, binge eating and emotional eating in post‐bariatric surgery women [21] and decreased sugar intake and fasting blood glucose levels among adults with obesity [22].

The MBES is based on the principles of mindfulness and intuitive eating and counteracts mindless eating. It aims to promote a healthy relationship with both the body and food [17, 23]. The protocol also encompasses body‐focused practices that promote body neutrality and body awareness through mindfulness and yoga, helping to foster body appreciation [24]. The MBES was applied in just one study, with women, and contributed to increased levels of intuitive eating, body appreciation and mindfulness, in which participants in the intervention group were more likely to have no symptoms for eating disorders compared to the waiting‐listed control group [17].

Most research involving MBIs has focused on clinical populations [12, 13, 25], including mindful eating interventions that have improved eating behaviours in individuals with overweight and obesity [26], binge eating disorder [26, 27] and people with problematic eating or body image concerns [28]. However, it is known that some nonclinical populations, such as nutrition students, are at high risk of developing eating disorders and disordered eating attitudes [5, 7] and could benefit from these interventions, such as shown by studies involving MBIs and student mental health [29, 30]. Therefore, the present study evaluated the effect of the online intervention MBES on the levels of body dissatisfaction and disordered eating attitudes of nutrition students during the COVID‐19 pandemic, with the hypothesis of improvement in these outcomes after the intervention.

## Methods

### Participants

Female undergraduate nutrition students enroled in 2020 in the universities of São Paulo, Brazil, aged 18 years and older and whohad access to the internet and electronic devices such as mobile phones or computers were included in the study. Moreover, individuals previously diagnosed with acute or chronic diseases that imposed some degree of dietary restrictions and/or psychopathologies (requiring the use of psychiatric drugs) to avoid the risk of adverse effects and emotional triggers [31] were excluded.

### Procedures

This randomised clinical trial was conducted using a parallel design with an active intervention group and a control group (no intervention). Due to the COVID‐19 pandemic, all procedures were conducted remotely through online platforms, specifically via synchronous videoconferencing sessions (e.g., Zoom). The study was approved by the Research Ethics Committee of the Hospital das Clínicas da Faculdade de Medicina de Ribeirão Preto da Universidade de São Paulo (opinions n.4.189.280 and n.4.395.551) and registered in the Brazilian Clinical Trials Registry (ReBEC: U1111‐1260‐1552).

The participants were recruited from August to September 2020 by advertising in social media platforms and sending institutional emails. The eligible participants were randomised to either the intervention group or the control group; the randomisation was performed by one of the researchers (first author), without blinding of both parties, using MedCalc, version 19.5.1 (MedCalc Software Ltd., Ostend, Belgium), with blocks stratified based on their body mass index (BMI) classification and availability to participate in the groups.

### Intervention

After the baseline assessment, the intervention group participated in the MBES [17, 23] performed by a nutritionist, an experienced instructor in this protocol. The MBES is characterised by a group intervention for 11 weeks, with weekly sessions lasting an average of 135 min. The MBES is based on the approach of specific themes such as mindfulness, intuitive eating, body acceptance and self‐care [23] and the promotion of formal mindfulness or mindful eating practices (Supporting Information S1: Chart 1). Body acceptance and self‐care involve a body‐neutral approach by embracing an instrumental perspective of the body, focusing on its functionality and value, rather than an appearance‐centred viewpoint [17, 20]. This protocol was adapted to Portuguese by the members of The Brazilian Center for Mindful Eating in collaboration with the original author. It was chosen for the study as it aligns with the concepts that help improve the outcomes assessed.

The protocol adopts the ‘**BASICS**’ of mindful eating, an acronym that enables the cultivation of the mindful eating skill. It includes **B**reathe and belly check for hunger and satiety before you eat, **A**ssess your food, **S**low down, **I**nvestigating hunger and satiety during meals, **C**hew your food thoroughly and **S**avor you food [17].

The participants received a manual of the MBES (with theoretical and practical content of each session) and audio recordings related to the formal mindfulness practices, which were sent using a mobile app. The participants were instructed to perform the formal mindfulness practices daily, to read about the topic discussed (MBES manual) and practise the ‘BASICS’ of mindful eating weekly.

From October to December 2020, two interventions were held in parallel on separate days (Tuesdays, *n* = 14 and Thursdays, *n* = 24) through videoconferences using the Zoom software (version 5.8.0, Zoom Video Communications Inc., California, USA). The frequency of the students’ participation during the sessions was monitored based on the attendance list and the videos recorded using a camera. Only two absences were allowed, which were accepted if the participant watched the recorded class and produced a report.

Two follow‐up sessions were conducted remotely via synchronous videoconferencing on the Zoom platform, 1 and 3 months after the intervention (January 2021 and April 2021). These sessions, also lasting approximately 135 min, were led by the same instructor and provided a review of previously covered concepts and practices, along with additional support for maintaining mindfulness practices. The average attendance during the follow‐up sessions was 25 participants.

Throughout the study period, two researchers (T.A.S. and T.S.B.) kept in touch with the participants using a mobile phone application to clarify any doubts and send reminders about the sessions, activities and evaluations at each time. However, the participants did not record their adherence to the tasks performed at home.

The control group did not receive any intervention. However, after the study was completed, participants in this group were given the opportunity to undergo the same protocol, including the follow‐up sessions.

### Outcome Assessment

**Figure 1:**
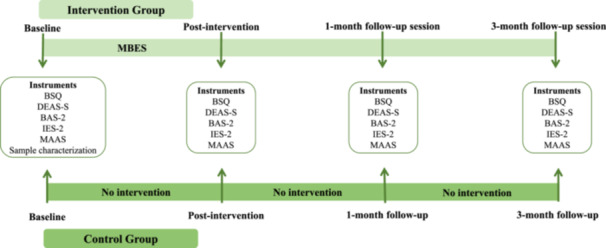
Study design. BSQ: Body Shape Questionnaire to assess body dissatisfaction; DEAS‐s: brief version of the Disordered Eating Attitude Scale to assess disordered eating attitudes; BAS‐2: Body Appreciation Scale‐2 to assess body appreciation; IES‐2: Intuitive Eating Scale‐2 to assess Reliance on Hunger and Satiety Cues and Body–Food Choice Congruence; MAAS: Mindful Attention Awareness Scale to assess mindfulness.

The participants of both groups were evaluated at four‐time points: at the beginning of the study (baseline), after the MBES group intervention (post‐intervention), at the 1‐month follow‐up and the 3‐month follow‐up, with a total duration of 6 months (Figure 1). Body dissatisfaction and disordered eating attitudes were evaluated as primary outcomes, whereas body appreciation, intuitive eating and mindfulness were evaluated as secondary outcomes. Instruments translated and validated for the Brazilian population were used for these evaluations.

### Sample Characterisation

The following data were collected using an online questionnaire: age (years), weight (kg) and height (m) reported for calculating and classifying the BMI (kg/m²) (underweight: < 18.5; normal weight: ≥ 18.5 to < 25; overweight: ≥ 25 to < 30; obesity: ≥ 30) [32], type of educational institution (private or public), the current level in undergraduate studies (first, second, third, fourth or fifth year), monthly income (less than half of the minimum wage, half to one time the minimum wage, 1–1.5 times the minimum wage, 1.5–2.5 times the minimum wage, more than 2.5 times the minimum wage or did not report), using the minimum wage reported in 2020 in Brazil (R$1045.00) as a basis.

### Body Dissatisfaction

The Body Shape Questionnaire (BSQ) translated and validated in Brazilian college students [33] was used to assess the degree of concern about body shape. The questionnaire contains 34 questions which were rated on a scale of 1 (*never*) to 6 (*always*). The total score was made by adding the scores for each item [33].

### Disordered Eating Attitudes

Disordered eating attitudes were assessed by the short form of the Disordered Eating Attitude Scale (DEAS‐s) validated in Brazilian college students [34].

The unidimensional scale has 17 items, and the total score was obtained by summing the scores for each item. Higher scores indicate more significant dysfunctional attitudes [34].

### Body Appreciation

The translated and validated version of the Body Appreciation Scale‐2 (BAS‐2) was used to assess the positive attitudes toward body image among Brazilian college students [35]. The scale contains 10 items that were rated on a scale of 1 (*never*) to 5 (*always*). The total score was obtained by summing up the scores on all items; higher scores reflect a greater body appreciation [35].

### Intuitive Eating

To assess the presence of intuitive eating attitudes and behaviours, the Intuitive Eating Scale‐2 (IES‐2) was used in its translated and validated version for Brazilian university students [36]. The IES‐2 consists of 18 items rated from 1 (*strongly disagree*) to 5 (*strongly agree*) and includes four subscales: (1) unconditional permission to eat (UPE), (2) eating for physical rather than for emotional reasons (EPR), (3) reliance on hunger and satiety cues (RHSC) and (4) body‐food choice congruence (B‐FCC) [36]. Higher scores indicate higher levels of intuitive eating.

### Mindfulness

To assess individual differences in the frequency of mindful states over time, the Mindful Attention Awareness Scale (MAAS) was used in its translated and adapted version for Brazilians [37]. The MAAS is a self‐administered scale with 15 items that are rated from 1 (*almost always*) to 6 (*almost never*). Higher scores indicate higher levels of mindfulness [37].

### Statistical Analysis

The Kolmogorov–Smirnov test was used to analyse the distribution of variables. Categorical variables were expressed as relative frequencies, whereas continuous variables were expressed as mean and standard deviation or error. The Mann–Whitney test was used for continuous variables and the chi‐square test for categorical variables to estimate the differences in sample characterisation data between the groups. Internal consistency reliability of scales and subscales was evaluated by Cronbach's alpha. Values > 0.70 were considered appropriate [38].

An a priori sample size calculation was conducted using GPower software (version 3.1.9.7). Repeated measures MANOVA *F*‐test was employed to compare two groups across four‐time points, applying a 5% alpha error probability, 80% of power, a conservative standard effect size of 0.25 and a correlation of 0.5 between repeated measures. This calculation indicated a required total sample size of *n* = 28 participants. To account for potential attrition, an additional 10%–20% was added, resulting in a final sample size of 31–34 participants.

However, recognising that the actual effect size for the outcomes and the actual correlation between measures might differ from the initial estimates, we conducted post hoc analyses to assess the final observed power. Since the outcome variables were not normally distributed, the effect size for the primary outcomes (body dissatisfaction and disordered eating) was estimated using the Wilcoxon signed‐rank test (*z*/√*n*). For baseline/post‐intervention comparisons, effect sizes of 0.49 and 0.51 were obtained, with correlations between repeated measures of 0.602 and 0.310, respectively.

These values, when considering a post hoc MANOVA *F*‐test for repeated measures between factors (two groups across four time points) with a 5% alpha error probability within factors, indicated an observed power of > 0.80 for the primary endpoints. Moreover, similar power levels were observed for other instruments across multiple time‐point comparisons, demonstrating the adequacy of the sample size for the analyses performed.

For details on effect sizes, correlations and observed power for each paired time‐point comparison, please refer to Supporting Information S2: Table 1.

All participants randomised for the study were analysed, regardless of their level of adherence. Since the intervention depended on participation in mindful eating sessions, the outcomes of volunteers who dropped out could not be assessed. Therefore, following literature criteria [39], we conducted the study using an intention‐to‐treat (ITT) analysis to avoid bias. As our sample was classified as missing completely at random (MCAR) according to Little's MCAR test (*p* = 0.804), multiple imputations were applied to handle missing data. We chose this method because it utilises available information to generate multiple plausible values, preserving statistical power and minimising bias by incorporating the uncertainty associated with missing values while maintaining data variability [39].

Due to the asymmetric distribution of the outcomes, Generalised Estimating Equations (GEEs) were employed to analyse continuous outcomes across groups (intervention and control) and time points. GEE is a statistical method for evaluating associations between repeated measurements within individuals in longitudinal studies [40].

All analyses were conducted using the Statistical Package for the Social Sciences‐IBM SPSS Statistics for Windows, version 20.1 (IBM Corp., Armonk, NY, USA), with a 95% confidence interval and a significance level set at *p* ≤ 0.05.

## Results

**Figure 2:**
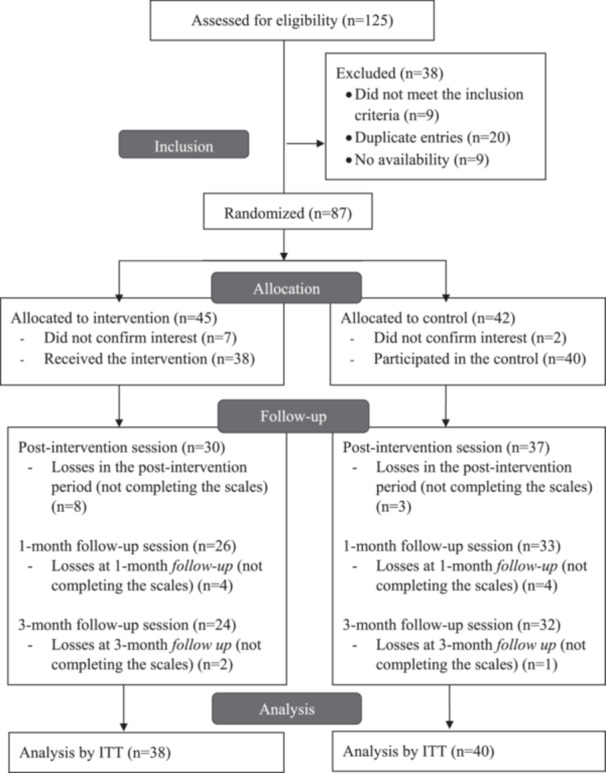
The flow of participants throughout the study.

Seventy‐eight randomised students took part in the study, between the intervention (*n* = 38) and control (*n* = 40) groups (Figure 2). A total of 14.1% (*n* = 11) of the participants from the intervention and control groups were lost during the intervention period. In the intervention group, 16 (42.1%) participants had 100% adherence to the 11 live sessions. Ten (26.3%) and four (10.5%) participants were absent, in one and two sessions, respectively, and later watched the recording session.

Good reliability was observed for the following scales: BSQ (intervention group: 0.97; control group: 0.96), DEAS‐s (intervention group: 0.82; control group: 0.81), BAS‐2 (intervention group: 0.93; control group: 0.93), MAAS (intervention group: 0.92; control group: 0.91), IES‐2 subscale RHSC (intervention group: 0.87; control group: 0.89) and IES‐2 subscale B‐FCC (intervention group: 0.83; control group: 0.70). However, data on the total IES‐2 scale score (intervention group: 0.48; control group: 0.60), IES‐2 EPR subscale (intervention group: −0.79; control group: −0.17) and UPE subscale (intervention group: 0.86; control group: 0.59) were not present due to low reliability.

### Sample Characterisation

The sample characterisation data were similar between the intervention and control groups at baseline (Table 1). The mean ages of participants in the intervention and control groups were 26.3 ± 10.0 and 24.8 ± 6.4 years, respectively. At baseline, weight (intervention group: 62.6 ± 12.5 kg; control group: 62.0 ± 10.0 kg) and BMI (intervention group: 23.6 ± 4.5 kg/m²; control group: 23.7 ± 3.8 kg/m²) were similar between the groups. About the BMI classification, the prevalence of normal weight was 73.7% (intervention group; *n* = 28) and 60.0% (control group; *n* = 24).

**Table 1:** Demographic, clinical and anthropometric characteristics of volunteers according to groups (*n* = 78).

Variables	Total (*n* = 78)	Intervention group (*n* = 38)	Control group (*n* = 40)	*p* value
Age (years), mean (SD)	25.6 (8.3)	26.3 (10.0)	24.8 (6.4)	0.988^a^
Type of educational institution, *n* (%)				
Public	30 (38.5)	17 (44.7)	13 (32.5)	0.267^b^
Private	48 (61.5)	21 (55.3)	27 (67.5)	
Level in undergraduate (years), *n* (%)				
First	7 (9.0)	2 (5.3)	5 (12.5)	0.787^b^
Second	29 (37.2)	14 (36.8)	15 (37.5)	
Third	15 (19.2)	7 (18.4)	8 (20.0)	
Fourth	22 (28.2)	12 (31.6)	10 (25.0)	
Fifth	5 (6.4)	3 (7.9)	2 (5.0)	
Monthly income (the minimum wage), *n* (%)				
< 0.5	2 (2.6)	1 (2.6)	1 (2.5)	0.214^b^
0.5– 1	7 (9.0)	2 (5.3)	5 (12.5)	
1– 1.5	12 (15.4)	4 (10.5)	8 (20.0)	
1.5–2.5	19 (24.4)	12 (31.6)	7 (17.5)	
> 2.5	27 (34.6)	11 (28.9)	16 (40.0)	
Did not report	11 (14.4)	8 (21.1)	3 (7.5)	
Weight (kg), mean (SD)	62.3 (11.2)	62.6 (12.5)	62.0 (10.0)	0.976^a^
BMI (kg/m^2^), mean (SD)	23.7 (4.1)	23.6 (4.5)	23.7 (3.8)	0.466^a^
Underweight *n* (%)	3 (3.8)	0 (0)	3 (7.5)	0.277^b^
Normal weight *n* (%)	52 (66.7)	28 (73.7)	24 (60.0)	
Overweight *n* (%)	15 (19.2)	6 (15.8)	9 (22.5)	
Obesity *n* (%)	8 (10.3)	4 (10.5)	4 (10.0)	

*Note:* Underweight: < 18.5 kg/m²; normal weight: ≥ 18.5 to < 25 kg/m²; overweight: ≥ 25 to < 30 kg/m²; obesity: ≥ 30 kg/m².

Abbreviation: BMI, body mass index.

^a^ Mann–Whitney test.

^b^ Chi‐square test.

### Primary and Secondary Outcomes

The baseline mean scores of the outcomes were similar between the intervention and control groups (*p *> 0.05) (Table 2). A significant decrease in body dissatisfaction was observed after the intervention (baseline: 86.6 ± 6.1 vs. post‐intervention: 64.1 ± 2.8; *p* < 0.05) and maintenance over time (1‐month follow‐up: 68.0 ± 3.3; 3‐month follow‐up: 67.2 ± 3.5). In the control group, there were no changes in the level of body dissatisfaction. The scores for body appreciation (BAS‐2) were similar between the two time points for both groups (Table 2).

**Table 2:** Model effect comparisons and post hoc tests of the variables at baseline and follow‐up, and findings using Generalised Estimating Equations (*n* = 78).

Variables	IG vs. CG	Intervention group (*n* = 38)				Control group (*n* = 40)				*p* value		
		Baseline	Post‐intervention	1‐Month follow‐up	3‐Month follow‐up	Baseline	Post‐intervention	1‐Month follow‐up	3‐Month follow‐up	Group	Time	Time × Group
	*p* value baseline	M (SD)	M (SD)	M (SD)	M (SD)	M (SD)	M (SD)	M (SD)	M (SD)			
Body dissatisfaction (BSQ)	0.997	86.60 (6.13)^a.b.c^	64.13 (2.82)^a^	68.00 (3.38)^b^	67.28 (3.51)^c^	91.80 (5.40)	88.26 (4.71)	87.60 (4.54)	88.20 (4.56)	0.001	< 0.001	0.001
Disordered Eating Attitude (DEAS‐S)	0.996	1.46 (0.08)^a^	1.15 (0.02)^a.b.c^	1.32 (0.05)^b^	1.29 (0.04)^c^	1.46 (0.07)	1.40 (0.05)	1.44 (0.07)	1.50 (0.06)	0.033	< 0.001	< 0.001
Body Appreciation (BAS‐2)	0.985	3.88 (0.11)	4.10 (0.07)	4.07 (0.08)	4.06 (0.09)	3.73 (0.11)	3.70 (0.11)	3.80 (0.11)	3.79 (0.11)	0.041	0.238	0.113
Intuitive Eating (IES‐2 subscale RHSC)	0.400	3.68 (0.13)^a.b.c^	4.37 (0.08)^a^	4.21 (0.09)^b^	4.24 (0.07)^c^	3.27 (0.15)	3.66 (0.12)	3.63 (0.12)	3.55 (0.14)	< 0.001	< 0.001	0.503
Intuitive Eating (IES‐2 subscale B‐FCC)	0.993	4.28 (0.10)	4.24 (0.11)	4.35 (0.08)	4.31 (0.08)	4.14 (0.10)	4.07 (0.11)	3.97 (0.11)	4.05 (0.10)	0.042	0.934	0.475
Mindfulness (MAAS)	0.954	3.38 (0.16)^a.b^	3.94 (0.12)^a^	3.88 (0.10)^c^	4.16 (0.11)^b.c^	3.65 (0.15)	3.62 (0.15)	3.64 (0.15)	3.68 (0.15)	0.303	0.002	0.007
BMI, kg/m^2^	1.000	23.63 (0.73)	23.40 (0.67)	23.75 (0.64)	23.61 (0.61)	23.76 (0.59)	23.71 (0.61)	23.73 (0.56)	24.09 (0.54)	0.788	0.463	0.327

*Note:* Generalised Estimating Equation. ^a–c^equal letters represent significant differences (*p* < 0.05).

Abbreviations: BAS, Body Appreciation Scale‐2; B‐FCC, body‐food choice congruence; BMI, body mass index; BSQ, Body Shape Questionnaire; CG, control group; DEAS‐s, brief version of the Disordered Eating Attitude Scale; IES‐2, Intuitive Eating Scale‐2; IG, intervention group; MAAS, Mindful Attention Awareness Scale; RHSC, Reliance on Hunger and Satiety Cues.

The disordered eating attitudes significantly decreased after the intervention (1.46 ± 0.08 vs. 1.15 ± 0.02); however, the score was significantly increased in the first month of follow‐up (1.32 ± 0.05) but was maintained in the third month of follow‐up (1.29 ± 0.04). The scores for disordered eating attitudes in the control group remained the same (Table 2).

A significant improvement was observed in the subscale RHSC (baseline: 3.68 ± 0.13 vs. post‐intervention: 4.37 ± 0.08; *p* < 0.05), maintained at the first and third months of follow‐up (1‐month follow‐up: 4.21 ± 0.09 vs. 3‐month follow‐up: 4.24 ± 0.07) in the intervention group. No difference was observed in the subscale IES‐2 B‐FCC between the two groups across time.

The scores for mindfulness were maintained in the control group, whereas the scores significantly increased in the intervention group after the MBES intervention (baseline: 3.38 ± 0.16 vs. post‐intervention: 3.94 ± 0.12; *p* < 0.05), with a significant increase at 3‐month follow‐up (4.16 ± 0.11) compared with that at baseline (Table 2). None of the groups showed significant changes in the average BMI across the four time points.

## Discussion

The MBES intervention decreased body dissatisfaction and disordered eating attitudes while increasing reliance on hunger and satiety cues and mindfulness in nutrition students. These improvements were maintained at 1‐ and 3‐month follow‐ups, except for disordered eating attitudes, which increased in the first month, but still in lower level than baseline, and mindfulness, which showed further improvement in the third month. No significant changes were observed in body appreciation or the B‐FCC subscale of the IES‐2.

Body dissatisfaction significantly decreased after the MBES intervention and was maintained during the follow‐up period. These findings align with previous research on MBIs, which have demonstrated improvements in body dissatisfaction levels [41, 42]. This outcome may be attributed to the MBES intervention's emphasis on cultivating a nonjudgemental mindset and fostering awareness of how thoughts, actions and environmental factors influence the body [17, 23].

Body dissatisfaction is not only linked to physical and mental health impairments [43] but also carries professional implications. Nutrition students face societal pressures to maintain thin bodies [44, 45], which are often tied to their perceived professional credibility [46]. Therefore, addressing body dissatisfaction in this population is essential, as it can undermine their professional identity, reducing both their perceived efficacy as dietitians and their overall job satisfaction [5]. However, it is worth noting that it is not enough to reduce the symptoms of negative body image (which encompasses body dissatisfaction); promoting a positive body image is essential. Positive body image is a multifaceted construct that fosters appreciation, respect, celebration and honour for one's body [47].

Contrary to expectations, no significant changes in body appreciation levels were observed after applying the MBES (3.88 ± 0.11 vs. 4.10 ± 0.07). This could be associated with a negative association between body appreciation and BMI [35], as 73.7% of participants in our study were normal‐weight women. A prior study using the same protocol (MBES) reported significant improvements in body appreciation among women with overweight and obesity (2.82 ± 0.67 vs 3.53 ± 0.06) [17]. Similarly, another study with a MBI involving predominantly normal‐weight female undergraduate students (72.3%) also reported improvements in body appreciation levels from pre‐ to post‐test (3.48 ± 0.78 vs. 3.84 ± 0.90) [48]. Despite the MBES sessions addressing positive body image [17, 23], the high baseline levels of body appreciation may explain the lack of improvement in this variable following the intervention.

It is important to highlight that individuals can have high body appreciation and still experience body dissatisfaction, and interventions may affect positive and negative body image in different ways [47, 49]. Furthermore, although the BAS‐2 was translated and validated for Brazilian individuals, further exploration is recommended to examine how the scale items relate to the concepts of positive body image and body appreciation within the Brazilian cultural context, as national and cultural factors may influence these perceptions [35].

The MBES intervention promotes a decrease in disordered eating attitudes in our study. Similar results were found by Bush et al., who observed that women with overweight/obesity who participated in the MBES were 3.65 times more likely to be asymptomatic for disordered eating than the control group [17]. Other studies with mindfulness interventions in students have demonstrated positive effects in reducing psychopathology related to eating disorders [50, 51]. Similar to our results, the changes in eating attitudes have not been maintained in the follow‐up period [50, 51].

Dysfunctional eating behaviours are influenced by socio‐environmental factors, such as peer and family pressure, which predominantly affect women [52]. Nutrition students, a predominantly female group, share overlapping factors for the risk of disordered eating behaviours and eating disorders. Some individuals who pursue a degree in nutrition may already have prior experiences with disordered eating or have lived in a larger body, facing stigmatising experiences [53]. They experience pressure to serve as a model of healthy eating and maintain a thin body [44, 45]. Moreover, the nutrition course curriculum is largely weight‐centred, a perspective widely reinforced by nutrition and dietetics educators [5, 54]. It is noteworthy that learning and professional development are social processes, where individuals are shaped by and influence their social environments [55]. These dynamics could have significant implications for their professional identities and practices [5, 54].

Therefore, the deep‐rooted beliefs of the search for the ideal body propagated in society [56] and reinforced in the context experienced by nutrition students [57] further stimulate these behaviours, making long‐term changes difficult. Our results highlight the importance of implementing long‐term educational initiatives to improve disordered eating attitudes and revising curricula alongside weight‐centred approaches that focus on the nutritional characteristics of food, often neglecting the sociocultural and emotional aspects of eating [5]. These actions can positively impact students’ physical and mental health while also preparing them for a more inclusive and assertive professional practice.

The intervention group also showed improvements in reliance on hunger and satiety cues (IES‐2 subscale RSHC) after the MBES intervention, which were maintained in the first and the third month of follow‐up. In the study of Bush et al. [17], the levels of the RHSC subscale also increased after the MBES intervention. Some studies proposed that mindfulness training can stimulate the brain areas involved in interoception increasing awareness of the body's internal state [41]. Developing trust in the body as the primary guide for eating, rather than external rules, is one of the pillars of the intuitive eating approach. Nevertheless, the intervention proved ineffective in increasing the levels of B‐FCC, which represents the ability to align food choices with the body's needs and signals. It is already known that some people need more time to understand, integrate and practise non‐dieting, non‐weight normative approaches [58], such as intuitive eating and mindful eating, so these concepts become naturalised and can be incorporated into the long‐term routine. Understanding and experiencing this approach is essential for nutrition students and their professional practice. A recent systematic review and meta‐analysis demonstrated that intuitive eating interventions improve disordered eating behaviours, intuitive eating, quality of life, body image and body appreciation [59].

The MBES intervention promoted increased mindfulness levels in nutrition students. Increased mindfulness levels after MBIs were expected and corroborated with previous studies [42, 60, 61]. In their study of MBES intervention, Bush found improvement in mindfulness levels and showed that mindfulness was partially responsible for the increases in intuitive eating [17]. Previous studies suggested that mindfulness can influence awareness and nonjudgement of sensations, thoughts and automatic patterns, helping to improve automatic eating behaviours and decision making [15, 62]. In our study, the mindfulness levels improved at the third‐month follow‐up, and the literature points out that the benefits could be more efficient after more intensive mindfulness meditation training and a consistent daily meditation practice [62].

As expected, no changes were observed in the BMI classification since most participants were normal weight, and weight change is not one of the goals of the MBES. Studies that include mindful eating interventions and analyses related to this outcome reported moderate weight loss in people with overweight and obesity [63] but showed that such interventions had a greater effect on weight maintenance [63, 64] with no significant differences compared with the results of other conventional dietary programmes [63].

Non‐weight‐focused approaches, such as mindful eating and intuitive eating, have been associated with positive physical health outcomes (lower BMI, better diet quality and increased physical activity) and mental health outcomes (reduced disordered eating and depressive symptoms, and improved body image, self‐compassion and mindfulness) in diverse populations [65]. A recent study showed that weight‐inclusive podcasts improved body appreciation, intuitive eating and anti‐fat attitudes among nutrition students [66]. Therefore, it may also help reduce weight stigma in nutrition students beyond promoting better eating behaviours and body image‐related outcomes.

The barriers and facilitators to implementing these approaches in clinical practice by Canadian dietitians were recently investigated. The main barrier related to the professionals’ characteristics was the lack of competence (knowledge and skills) and confidence to apply these approaches, as their training is predominantly focused on weight‐centred methods [67, 68]. Thus, our findings highlight the need to explore mindful eating interventions as additional educational strategies in nutrition courses and curricula to improve students’ relationships with food and body image, as well as support their professional development.

The present study is relevant as it is the first to implement a mindful eating intervention protocol with nutrition students, a high‐risk group for disordered eating and eating disorders development. It could influence people's relationship with food and body image in their future practice. Moreover, it is the first randomised clinical trial conducted using the MBES, opening new possibilities for intervention and research in other populations with eating and weight concerns.

This study showed positive results after the MBES intervention in female nutrition students, decreasing the levels of body dissatisfaction, and disordered eating attitudes, and increasing the levels of intuitive eating (reliance on hunger and satiety cues subscale) and mindfulness, thus assisting in the management of sociocultural influences on body image and eating behaviours in this group. It is possible that emotional regulation, self‐compassion and interoceptive awareness of hunger and fullness cues, associated with the increased mindfulness levels [69], and a possible reduction in thin‐ideal internalisation [70], may have contributed to these effects. Exploring these potential mediators in future studies may help better understand the underlying mechanisms of the intervention's effectiveness.

These findings must also be interpreted within the context of the study's limitations. Initially, we highlight the non‐blinding of researchers and study participants. Participants were virtually recruited from universities in São Paulo (Brazil) and randomised into intervention and control groups, minimising the risk of contamination as intervention participants were unaware of control group identities. However, potential selection bias may have arisen due to the sample's likely health‐conscious and research‐interested nature. Additionally, ‘social desirability’ bias may have influenced participants to provide more socially acceptable responses [71]. The 11‐week programme, the two follow‐up meetings, home activities and the challenges of the COVID‐19 pandemic, including remote work and study demands, fatigued some participants, affecting adherence. It is worth noting that students received no monetary incentives to participate in the intervention. Additionally, the absence of validated instruments to assess mindful eating in Brazilian Portuguese was a limitation.

As this preliminary study involves a mindful eating intervention among nutrition students with a waitlist control group, it does not allow for determining the superiority of MBES over other interventions. This underscores the need for future studies employing active control groups for more robust comparisons with established treatments for disordered eating and body dissatisfaction (such as using cognitive behavioural therapy, acceptance and commitment therapy, self‐compassion training, supportive counselling, relaxation training programmes, general stress management programmes, exercise‐based interventions, psychoeducation programmes or health promotion interventions). We also suggest measuring the frequency and duration of guided mindfulness practices performed at home between sessions to gain a better understanding of adherence to the protocol.

## Author Contributions

All authors contributed to the study conception and design. **Taísa Alves Silva:** conceptualization, investigation, methodology, formal analysis, writing – original draft, writing – review and editing, data curation. **Amanda Thaís Flôres and Thamara Smaniotto Buttros:** conceptualization, investigation, methodology, formal analysis, writing – review and editing. **João Henrique Fabiano Motarelli:** investigation, methodology, resources, writing – review and editing. **Geórgia das Graças Pena:** methodology, formal analysis, writing – review and editing. **Fernanda Rodrigues de Oliveira Penaforte:** resources, formal analysis, writing – review and editing. **Camila Cremonezi Japur:** conceptualization, supervision, methodology, project administration, funding acquisition, writing – review and editing.

## Ethics Statement

The study was approved by the Research Ethics Committee of the Hospital das Clínicas da Faculdade de Medicina de Ribeirão Preto da Universidade de São Paulo (opinions n.4.189.280 and n.4.395.551). All participants completed the informed consent form.

## Conflicts of Interest

The authors declare no conflicts of interest.

### Peer Review

The peer review history for this article is available at https://www.webofscience.com/api/gateway/wos/peer-review/10.1111/jhn.70040.

## Transparency Declaration

The lead author affirms that this manuscript is an honest, accurate, and transparent account of the study being reported. The reporting of this work is compliant with CONSORT guidelines. The lead author affirms that no important aspects of the study have been omitted and that any discrepancies from the study as planned (registered in the Brazilian Clinical Trials Registry: U1111‐1260‐1552) have been explained.

## Supporting information

Supporting information.

Supporting information.

## Data Availability

The data that support the findings of this study are available from the corresponding author upon reasonable request.
