# Supplementary material for: Mindfulness‐Based Eating Solution (MBES) for Body Dissatisfaction and Disordered Eating Attitudes in Nutrition Students During the COVID‐19 Pandemic: A Randomised Clinical Trial
Source: J Hum Nutr Diet. 2025 Mar 17;38(2):e70040. doi: 10.1111/jhn.70040 (PMC11911907; doi:10.1111/jhn.70040)
Supplement: Supplementary file 1 — Supporting information. [file JHN-38-0-s001.docx]

**Chart 1 - Supplementary material.** Topics addressed in the Mindfulness-Based Eating Solution (MBES), with an average duration of 135 minutes in each meeting.

| **Meetings** | **Topics covered** | **Exercises in the meeting** | **Homework** |
| --- | --- | --- | --- |
| 0 | Introduction to the MBES: theoretical basis, review of each week’s topics | Practice mindfulness of breath meditation. | Practice mindfulness of breath meditation. |
| 1 | BASICS of mindful eating: breathe and belly check for hunger and satiety before you eat; assess your food; slow down; investigate your hunger throughout the meal; chew your food thoroughly; savor your food. | Practice mindfulness of breath meditation.  Improve practices. | Practice mindfulness of breath meditation.  Use BASICS at mealtimes.  Read the tips and guidelines in the manual. |
| 2 | Talking to the body (what is your body telling you, and what are you telling your body?) | Practice body scanning and show loving kindness to the body | Practice mindfulness of breath meditation or body scanning.  Use BASICS and hunger scales at mealtimes.  Pay attention to the body signals that indicate what it needs (food, movement, etc.).  Read the tips and guidelines in the manual. |
| 3 | Food Wisdom (balancing the Three Food Wisdoms by (a) no forbidden food, (b) eating the “right amount” of food, and (c) knowing and respecting your habitsand triggers about food) | Show loving kindness to the body  Practice mindfulness of breath meditation | Practice mindfulness meditation during breathing or show loving kindness to your body.  Use BASICS and hunger scales at mealtimes  Read the tips and guidelines in the manual. |

**Chart 1 - Supplementary material.** Topics addressed in the Mindfulness-Based Eating Solution (MBES), with an average duration of 135 minutes in each meeting. (continuation)

| **Meetings** | **Topics covered** | **Exercises in the meeting** | **Homework** |
| --- | --- | --- | --- |
| 4 | Food thoughts: Know what they are and how to listen to them. | Practice mindfulness of thoughts. | Practice mindfulness of thoughts or show loving kindness to your body.  Use BASICS and hunger scales at mealtimes.  Read the tips and guidelines in the manual. |
| 5 | What are you really (really) hungry for? Do not use food to resolve emotions. | Practice self-compassionate touch. | Practice mindfulness of the breath or show loving kindness to your body or practice body scanning.  Use BASICS and hunger scales at mealtimes.  Recognize the types of famines.  Read the tips and guidelines in the manual. |
| 6 | Supporting satiety and satisfaction: Know the types of foods that support a feeling of satiety and satisfaction. | Practice mindful yoga. | Practice mindfulness of breath or mindful yoga  Use BASICS and hunger and satiety scales at mealtimes.  Read the tips and guidelines in the manual. |
| 7 | Becoming physically active (finding ways to become more physically active) | Practice walking with full attention. | Practice mindfulness of breath or mindful yoga.  Use BASICS and hunger and satiety scales at mealtimes.  Read the tips and guidelines in the manual. |

**Chart 1 - Supplementary material.** Topics addressed in the Mindfulness-Based Eating Solution (MBES), with an average duration of 135 minutes in each meeting. (Continuation)

| **Meetings** | **Topics covered** | **Exercises in the meeting** | **Homework** |
| --- | --- | --- | --- |
| 8 | Becoming a conscious knower | Taste test (ultra-processed versus fresh/minimally processed foods) | Practice mindfulness of breath, body scanning, loving kindness to your body, or mindful yoga.  Use BASICS and hunger and satiety scales at mealtimes.  Read the tips and guidelines in the manual. |
| 9 | Respecting and appreciating your body | Practice movement (yoga and slow walking). | Practice mindfulness of breath, body scanning, loving kindness to your body, or mindful yoga.  Use BASICS and hunger and satiety scales at mealtimes.  Read the tips and guidelines in the manual;  Complete the food journal. |
| 10 | “Eat for Life” (continuing the path of Mindful Eating). | Practice movement (yoga and slow walking).  Practice mindfulness of breath. | Answer the questionnaires at the end of the meeting. |

Source: Adapted from Rossy, 2016.

The control group did not receive any type of ‘usual care’ or education sessions.
